# Molecular Classification of Patients With COVID‐19 Based on Transcriptional Profiling

**DOI:** 10.1111/irv.70227

**Published:** 2026-02-10

**Authors:** Hongyu Liu, Ying Zheng, Xiaoyan Deng, Mengxue Li, Di He, Wenting Zuo, Yitian Xu, Xuhui Shen, Haibo Li, Bin Cao

**Affiliations:** ^1^ Department of Pulmonary and Critical Care Medicine, China–Japan Friendship Hospital Capital Medical University Beijing People's Republic of China; ^2^ National Center for Respiratory Medicine, State Key Laboratory of Respiratory Health and Multimorbidity National Clinical Research Center for Respiratory Diseases, Institute of Respiratory Medicine, Chinese Academy of Medical Sciences & Peking Union Medical College, Department of Pulmonary and Critical Care Medicine, New Cornerstone Science Foundation, Center of Respiratory Medicine, China‐Japan Friendship Hospital Beijing People's Republic of China; ^3^ Tsinghua University‐Peking University Joint Center for Life Sciences Beijing People's Republic of China; ^4^ Laboratory of Clinical Microbiology and Infectious Diseases, Department of Pulmonary and Critical Care Medicine, National Center for Respiratory Medicine, China–Japan Friendship Hospital Beijing People's Republic of China; ^5^ Institute of Respiratory Medicine Chinese Academy of Medical Sciences Beijing People's Republic of China; ^6^ Peking University China–Japan Friendship School of Clinical Medicine Beijing People's Republic of China; ^7^ National Center for Respiratory Medicine Beijing People's Republic of China; ^8^ State Key Laboratory of Respiratory Health and Multimorbidity Beijing People's Republic of China; ^9^ National Clinical Research Center for Respiratory Diseases Beijing People's Republic of China; ^10^ Department of Pulmonary and Critical Care Medicine Center of Respiratory Medicine, China–Japan Friendship Hospital Beijing People's Republic of China

**Keywords:** COVID‐19, precision treatment, SARS‐CoV‐2, transcriptome

## Abstract

**Background:**

COVID‐19 has caused over 7 million deaths worldwide and remains a critical public health threat. The marked heterogeneity in immune responses among patients poses challenges for targeted treatment. Molecular classification is essential for guiding precision therapies.

**Methods:**

We performed unsupervised consensus clustering on blood transcriptomic data from 351 COVID‐19 patients to identify molecular endotypes and validated the classification in an independent cohort of 56 patients. To identify robust endotype‐specific biomarkers, we applied XGBoost, LASSO, and random forest algorithms.

**Results:**

Three endotypes with distinct biological and clinical profiles were identified. Endotype 1, associated with favorable outcomes, showed enriched DNA replication pathways and elevated IL7 expression. Endotype 2 featured hypoxia and angiotensin‐related pathways. Endotype 3 exhibited TLR4 activation, IL‐1β upregulation, and impaired NK cytotoxicity, correlating with poor outcomes. All endotypes shared type I interferon activation. Predictive biomarker pairs included STAT4:S100A11 (endotype 1), SLC4A1:RPL31 (endotype 2), and RALB:MTR (endotype 3), enabling endotype classification with high accuracy. Importantly, these biomarker genes can be reliably measured in peripheral blood using RT‐qPCR, making the classification model feasible for clinical application.

**Conclusions:**

This molecular classification reveals heterogeneity in COVID‐19 and proposes biomarker‐guided strategies for patient stratification and management.

## 1 Introduction

The COVID‐19 pandemic, caused by severe acute respiratory syndrome coronavirus 2 (SARS‐CoV‐2), has resulted in substantial morbidity and mortality. As of December 2023, over 700 million cases of infection and 7 million deaths had been reported globally [1]. Although COVID‐19 mortality rates have decreased, new cases continue to be confirmed worldwide, and COVID‐19 remains a significant public health concern [2].

Several risk factors have been identified for COVID‐19, including older age, male sex, obesity, and the preexisting comorbidities. Beyond these clinical risk factors, severe COVID‐19 is characterized by a dysregulated immune response, notably marked by a cytokine storm, impaired type I interferon signaling, and altered activity of myeloid cells [3]. Blood transcriptional profiling has significantly advanced our understanding of SARS‐CoV‐2 pathogenesis [4, 5, 6, 7, 8, 9]. Single‐cell sequencing analysis of peripheral blood cells from COVID‐19 patients revealed a reduction in CD14^low^CD16^high^ nonclassical monocytes and an accumulation of HLA‐DR^low^ classical monocytes in severe cases [10]. Interferon (IFN) responses in critical COVID‐19 patients are characterized by a marked downregulation of interferon‐stimulated genes (ISGs), such as MX1, IFITM1, and IFIT2, indicating a compromised antiviral response [11]. Moreover, disease severity has been associated with elevated levels of pro‐inflammatory cytokines, including IL‐1β, IL‐6, TNF‐α, IL‐2, and IL‐7 [12, 13, 14]. Exacerbated cytokine production is the underlying cause of death in SARS‐CoV‐2‐infected individuals.

Although supervised analyses of COVID‐19 patients with discordant outcomes (i.e., survivors vs. non‐survivors) have identified candidate gene biomarkers [15, 16, 17], such as CD177 [4], substantial heterogeneity in treatment response remains unexplained. Existing predictive models for COVID‐19 subtypes have demonstrated limited generalizability and accessibility [18, 19]. Therefore, there is an urgent need for novel molecular classification approaches to facilitate precision treatment strategies for patients with COVID‐19.

Molecular characterization of diseases has contributed to improvements in clinical treatment strategies. Unsupervised learning has been successfully applied to assess heterogeneity in tumor patients, revealing distinct patient subtypes with unique biological and clinical characteristics [20, 21]. These findings suggest the potential for subtype‐specific therapeutic strategies. However, a comprehensive assessment of heterogeneity in COVID‐19 patients has not yet been conducted. Here, we analyzed RNA expression profiles to investigate the molecular characterization of COVID‐19.

## 2 Materials and Methods

### 2.1 Study Design

The transcriptional data of COVID‐19 patients were obtained from the Gene Expression Omnibus (GEO) database, including GSE152418, GSE152641, GSE157103, GSE161731, GSE171110, GSE179627, and GSE222393. The details of the seven datasets were summarized in Table S1. R package “sva” (v3.46.0) was applied to remove batch effects from combined data [22]. Our study includes two validation cohorts: a prospective cohort of 56 patients enrolled at the China–Japan Friendship Hospital between December 2022 and April 2024, and the publicly available dataset GSE217948. Patients were included if they had a confirmed SARS‐CoV‐2 infection, either by reverse transcriptase polymerase chain reaction (RT‐PCR) or an antigen test. All patients had written informed consent. This study was approved by the Ethics Committee of China–Japan Friendship Hospital (2022‐KY‐058). Demographic information is summarized in Table S3.

### 2.2 Sample Collection and Processing

A peripheral blood sample was collected from the patient and centrifuged using Ficoll‐Paque PLUS (Cytiva, Cat# 17144003). The peripheral blood mononuclear cells (PBMCs) were washed with PBS (Invitrogen), centrifuged, and preserved in Trizol (Invitrogen, Cat# 15596026) at −80°C. Bulk RNA‐seq was performed using the Illumina NovaSeq 6000 with a PE150 read length.

### 2.3 Consensus Clustering

Gene expression data performed unsupervised consensus clustering (R package ConsensusClusterPlus v.1.62.0) using the 5000 genes with the highest median absolute deviation (MAD), 1000 repetitions, 0.8 of subsampling for each repetition, and k between 2 and 10 [23]. R package “cluster” (v 2.1.6) was used to conduct Silhouette analysis [24].

### 2.4 Co‐Expression Network Construction

The top 50% (8471) of genes with the highest MAD used as a robust measure of variability were selected for WGCNA. Pearson correlation analysis between two genes is used to construct the similarity matrix. The adjacency matrix is calculated, and the topological overlap matrix (TOM) is constructed to describe the association strength between the genes. 1‐TOM was used as an input for the hierarchical clustering analysis of genes, and the cutreeDynamic function was applied to identify network modules. The co‐expression modules that meet the conditions (minModuleSize = 30, deepSplit = 2, height = 0.25).

### 2.5 Differentially Expressed Genes (DEGs) and Machine Learning

The DEGs analysis was performed using the “DESeq2” package [25] with the threshold value of |logFC| > 1 and adj.P.Val < 0.05. DEGs unique to each endotype (compared to all other endotypes) were used to identify key genes through machine learning. The “caret” package splits the dataset before building the classification model, using 70% of the samples as training data and 30% as test data. The XGBoost [26], LASSO [27], and random forest classifier [28] were used to establish the classification model and select key genes from the training data. Each model underwent tenfold cross‐validation, and the receiver operating characteristic (ROC) curve was plotted to calculate the area under the curve (AUC) on the test data using the “multiROC” package. The results were visualized using the R packages “ggplot,” “pheatmap,” and “VennDiagram.”

### 2.6 Endotype Biomarker Models Construction

All genes selected by machine learning were combined to calculate the two‐gene expression ratio (endotype score) [29]. The R package “pROC” (v 1.18.5) was used to calculate the “best” thresholds. Among all the two‐gene expression ratios, the classifier with the highest AUC was designated as the predictive model.

$$\text{endotype score}=\left[{\text{gene}}_{i}/{\text{gene}}_{j}\right]$$

### 2.7 Pathway Enrichment Analysis

Gene ontology (GO), Kyoto Encyclopedia of Genes and Genomes (KEGG) enrichment analyses, and gene set enrichment analysis (GSEA) of GO were performed using the R package “clusterProfiler” (version 4.6.2). The Benjamini–Hochberg method was used for the multiple corrections, and a false discovery rate (FDR) < 0.05 was considered significant.

### 2.8 Circulating Cell Proportions

The proportions of PBMCs in each sample were estimated using CIBERSORT [30]. Cell populations not commonly found in PBMCs were excluded from the original LM22 reference matrix.

### 2.9 Quantitative PCR

Total RNA was extracted from PBMCs using TRIzol reagent (Invitrogen, Cat# 15596018). Reverse transcription was performed using the RevertAid First Strand cDNA Synthesis Kit (Thermo Scientific, Cat# K1622). Quantitative real‐time PCR was carried out using the PowerUp SYBR Green Master Mix (Applied Biosystems, Cat# A25742) on the QuantStudio 5 Real‐Time PCR System (Applied Biosystems, Cat# A28569). Primer sequences used for qRT‐PCR are listed in Table S4. Gene expression levels were normalized to ACTB using the 2^−ΔΔCt^ method.

### 2.10 Statistical Analysis

Statistical analysis was done using the R statistical computing environment (version 4.2.3). Continuous variables were described as median (IQR) or mean ± standard deviation, depending on the normality of distribution. Continuous data were analyzed using the Wilcoxon test or Kruskal–Wallis test, as appropriate, while categorical data were compared using the chi‐square test or Fisher's exact test.

## 3 Results

### 3.1 Subtypes of COVID‐19 and Their Association With Clinical Feature

**FIGURE 1:**
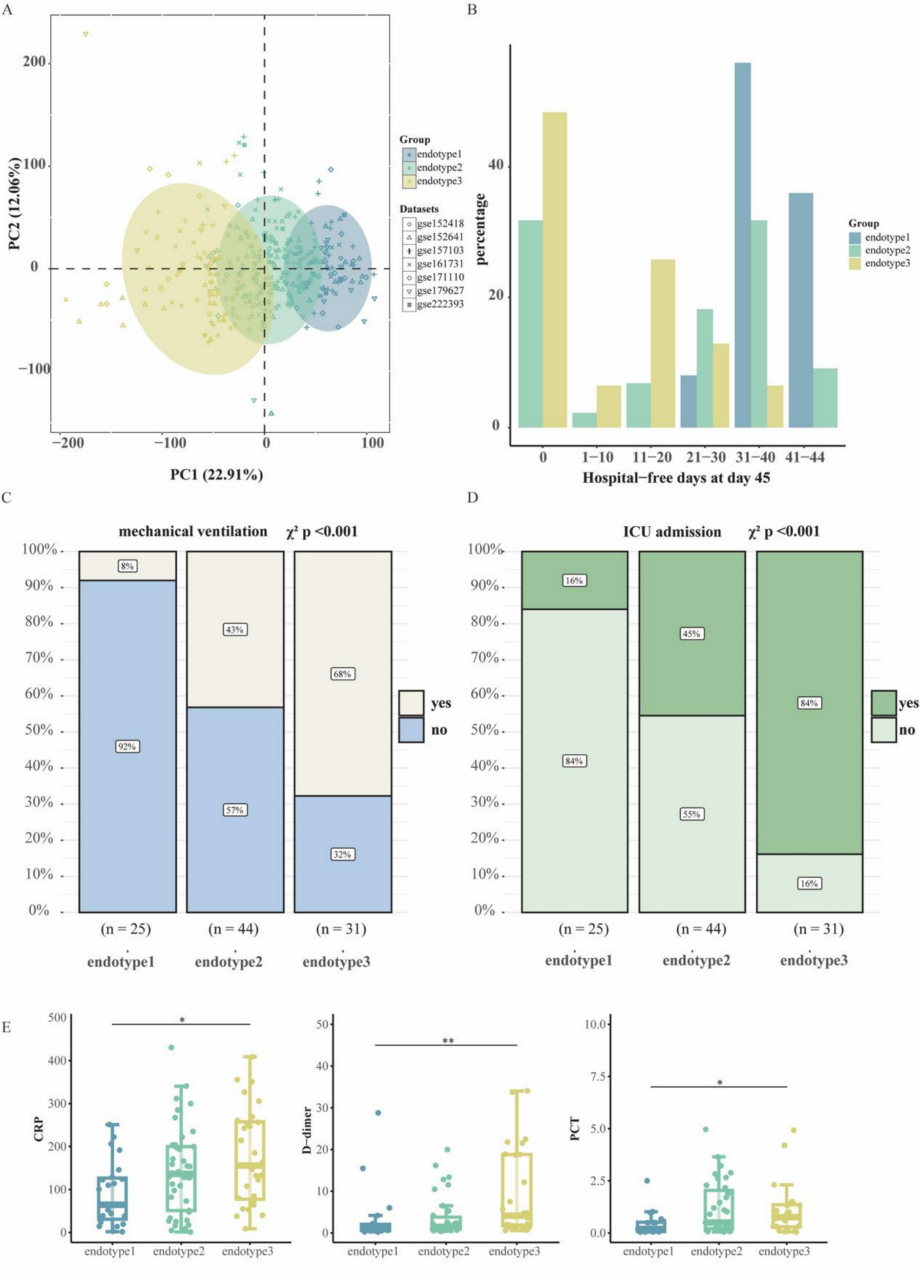
Subtypes of COVID‐19 and their clinical outcomes. (A) Principal component analysis demonstrating the separation of COVID‐19 subtypes based on gene expression profiles (*n* = 351). (B) Hospital‐free days within 45 days for each subtype group. (C, D) Comparison of mechanical ventilation (C) and ICU admission (D) proportions between each endotype group. (E) The laboratory measurements (CRP, D‐dimer, and PCT) in the endotypes. Two‐sided *p* values were calculated using the Wilcoxon test and adjusted with the Bonferroni method (*p < 0.05, **p < 0.01, ***p < 0.001, ****p < 0.0001, this labels also applies to the following figures).

A total of 351 COVID‐19 samples and 92 healthy control samples were included for the following analysis (Table S1). Consensus clustering identified three subtypes with distinct molecular and clinical features, designated as endotype 1–3 (Figure S1A). Convergent evidence from consensus clustering metrics [23] and NbClust [31] analysis consistently supported k = 3 as the best number of molecular subtypes (Figure S1A–E). The PCA plot showed the differences between the three endotypes (Figure 1A). To clarify the molecular features underlying the variation along PC1, we calculated the PC1 loadings and performed pathway enrichment analyses. The full results are summarized in Table S2. The clinical data from the GSE157103 dataset revealed COVID‐19 endotypes associated with disease severity. The outcome parameter “hospital‐free days at day 45” (HFD‐45) is assigned a value of zero for patients who are hospitalized for more than 45 days or who die during their hospitalization. Patients with shorter hospital stays receive higher HFD‐45 values. The endotype 3 group had a lower HFD‐45 score compared to the group endotype 1 and endotype 2 (Figure 1B). Additionally, the prevalence of COVID‐19 patients requiring mechanical ventilation was highest in endotype 3 (68%) and lowest in endotype 1 (8%) (chi‐squared test, *p* < 0.001; Figure 1C). Moreover, the endotype 3 group had the highest proportion of ICU admissions (chi‐squared test, *p* < 0.001; Figure 1D). Laboratory abnormalities observed in COVID‐19 have been reported, including elevated serum levels of C‐reactive protein (CRP), D‐dimer, and procalcitonin (PCT) [32]. CRP and PCT are inflammatory markers, while D‐dimer is associated with abnormal coagulation function. CRP, D‐dimer, and PCT tended to exhibit the highest levels in endotype 3 and the lowest levels in endotype 1 (Figure 1E). Collectively, these clinical features suggest that endotype 3 had the worst prognosis, while endotype 1 had the best outcomes.

### 3.2 Key Genes and Predictive Model for COVID‐19 Subtypes

**FIGURE 2:**
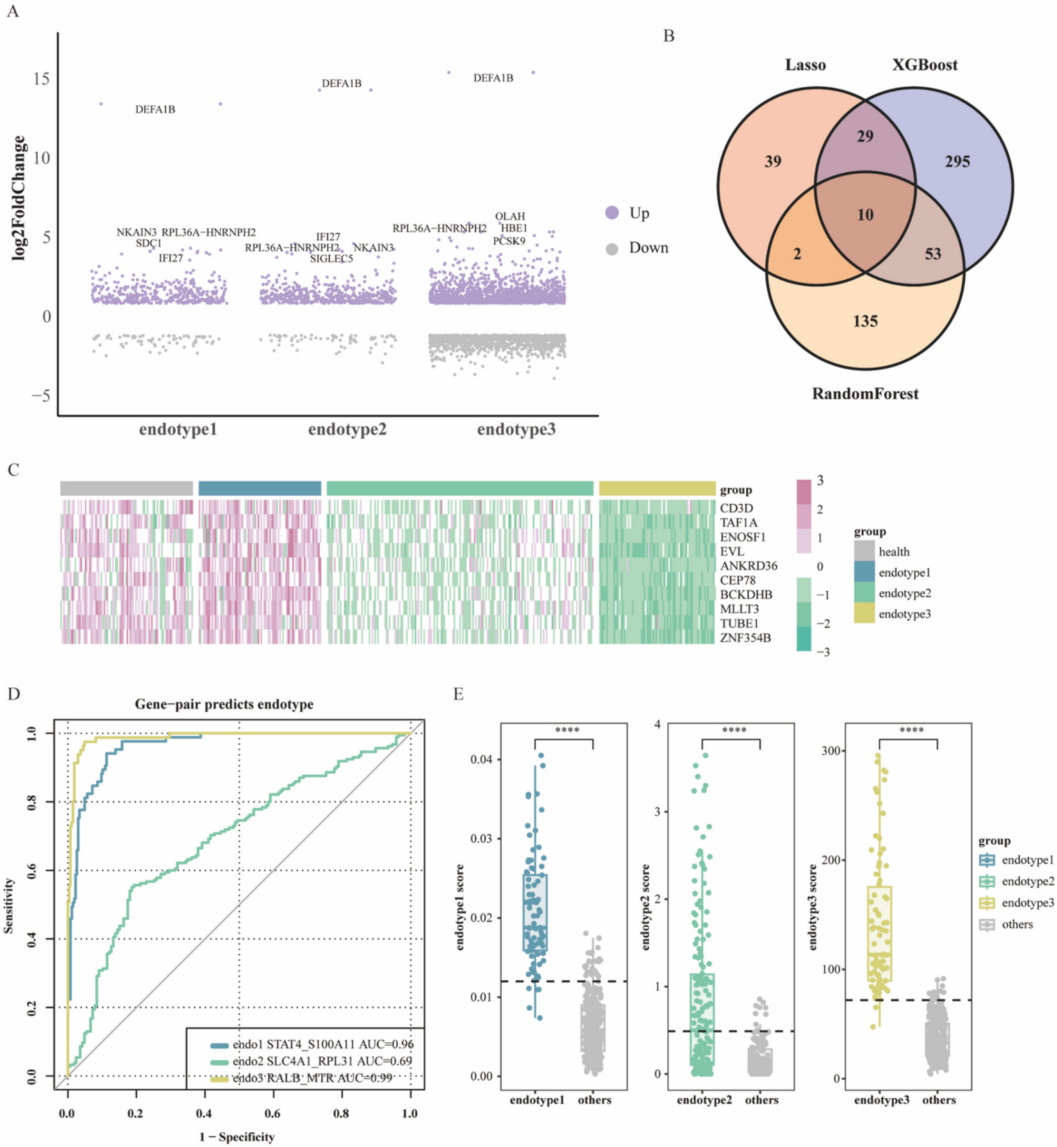
Differential expression analysis and key gene classification of COVID‐19 patient subtypes. (A) Volcano plot displays the differential gene expression in the endotype 1–3 groups (*n* = 351) compared with healthy individuals (*n* = 92). (B) Venn diagram showed the intersection of genes selected by three machine learning approaches. (C) The heatmap of intersecting genes. (D) ROC curves for using the gene expression ratio to identify the endotypes. (E) Box plot of the gene expression ratios (endotype score) used to discriminate different endotype. The horizontal line in the box plot denotes the threshold values: endotype 1 (0.012), endotype 2 (0.489), and endotype 3 (71.904). *p* values were from the two‐sided Wilcoxon test.

Differential gene expression analysis was performed for the three endotypes compared with the healthy control, with endotype 3 showing the highest number of unique DEGs (Figures 2A and S2A). The top 5 genes in each endotype group were labeled (Figure 2A). Three machine learning methods, including XGBoost, LASSO, and the random forest classifier, were employed to identify DEGs for classifying the three endotypes and constructing a predictive model (Figure 2B). The LASSO model had the highest AUC (0.991) (Figure S2B). The genes selected by each method were different, and there were 10 genes overlapping (Figures 2C and S2C). The 10 genes were ANKRD36, BCKDHB, CD3D, CEP78, ENOSF1, EVL, MLLT3, TAF1A, TUBE1, and ZNF354B (Figure 2C). To facilitate potential clinical applications, all genes selected by the three machine learning methods were utilized to construct candidate endotype biomarker models. The resulting models were as follows: STAT4:S100A11 for endotype 1 (threshold = 0.012, AUC = 0.96), SLC4A1:RPL31 for endotype 2 (threshold = 0.489, AUC = 0.69), and RALB:MTR for endotype 3 (threshold = 71.904, AUC = 0.99) (Figure 2D,E).

### 3.3 Biological Interpretation of the Three COVID‐19 Subtypes

**FIGURE 3:**
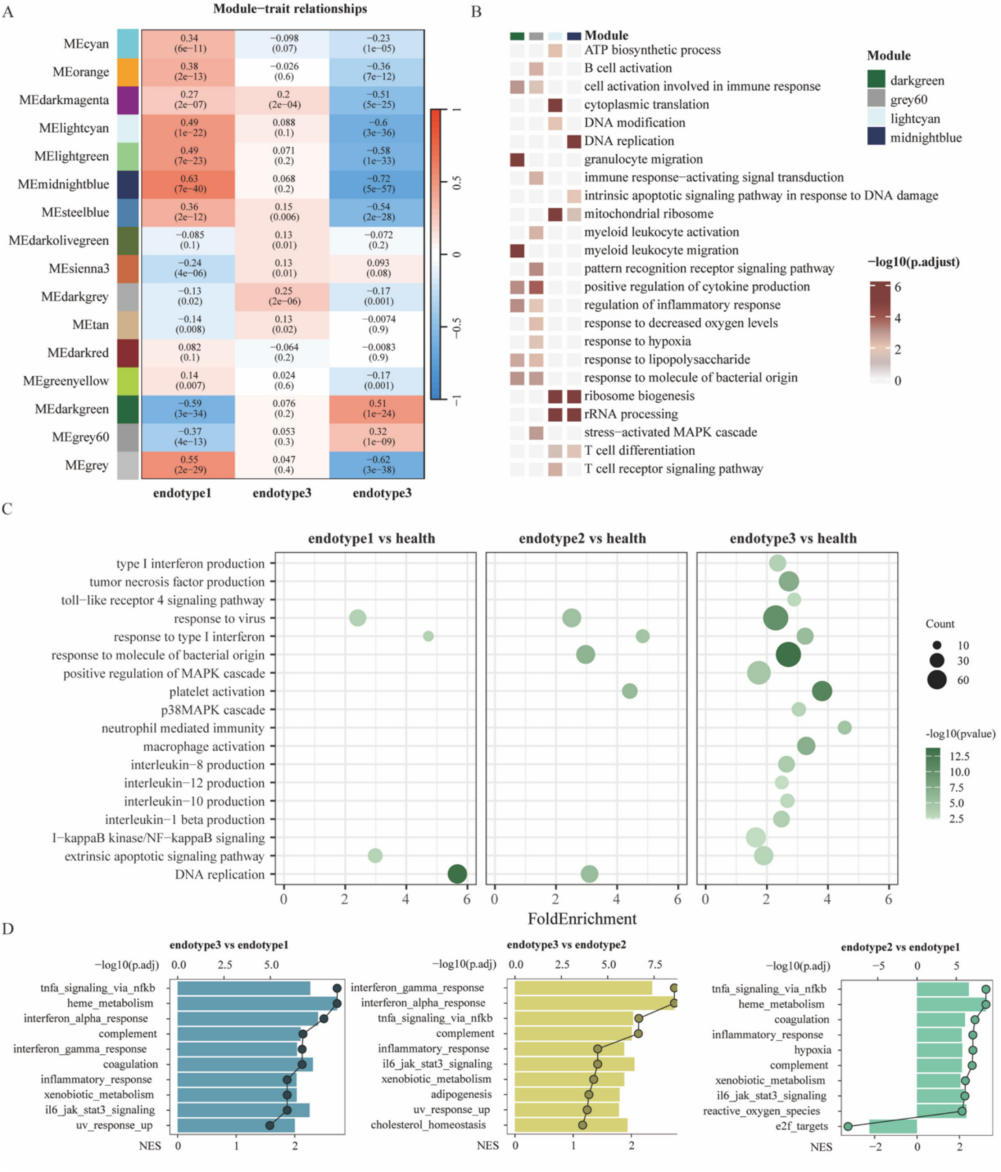
Biological function analysis of COVID‐19 subtypes. (A) Heatmap showing the correlation between module eigengenes and endotypes. (B) Functional enrichment analysis (GO) of modules associated with endotypes. (C) GO enrichment for upregulated DEGs in different endotype group. The values on the x‐axis were defined as the relative percentage of genes belonging to each pathway, divided by the corresponding percentage in the background. (D) Hallmark pathway analysis comparing the three endotypes. The top 10 enriched pathways are shown. Box plots represent normalized enrichment scores (NES), and dots indicate the –log10(adj. *p* value).

WGCNA analysis identified 16 modules (Figures 3A and S3A–C). Among these, the lightcyan and midnightblue modules showed positive correlations with endotype 1, whereas the darkgreen and grey60 modules were strongly associated with endotype 3 (Figure 3A). Modules enriched in endotype 3 were dominated by immune‐related programs, including the pattern‐recognition receptor signaling pathway and the stress‐activated MAPK cascade (Figure 3B). In contrast, pathways enriched in the lightcyan and midnightblue modules were mainly involved in ATP biosynthesis, ribosome biogenesis, and T cell receptor signaling (Figure 3B). Compared with healthy controls, all three endotypes demonstrated activation of type I interferon and antiviral response pathways. Endotypes 2 and 3 showed additional enrichment in responses to bacterial molecules and platelet activation. Endotype 3 displayed the strongest activation of immune‐associated pathways, including cytokine production, NF‐κB signaling, and TLR4 signaling, highlighting its highly inflammatory profile (Figure 3C). Endotype 1 was characterized by downregulation of IL1‐mediated signaling and upregulation of pathways related to DNA replication (Figures 3C and S3D). Neutrophil‐mediated immunity was increased in endotype 3 but suppressed in endotype 1 (Figures 3C and S3D). Endotype 2 was enriched for hypoxia‐related pathways and the angiotensin‐activated signaling pathway (Figure S3E). Notably, MHC class II protein complex assembly was consistently downregulated in endotypes 2 and 3 (Figure S3F,G).

To further delineate the drivers of immune activation across endotypes, we performed differential expression and Hallmark pathway analyses. CD177, a key regulator of neutrophil activation, was most highly expressed in endotype 3, in line with the pronounced neutrophil‐mediated immune response in this group (Figure S3H). As noted above, endotype 3 exhibited the strongest type I interferon response and inflammatory activity. Endotype 2 also displayed a greater inflammatory signature than endotype 1, including augmented IL6‐JAK‐STAT3 and TNFA signaling (Figure 3D). Finally, given that endotype 3 was associated with the poorest clinical outcomes, we further examined its unique biological features relative to the other endotypes. Compared with both endotypes 1 and 2, endotype 3 showed suppression of multiple key immune pathways, including the chemokine signaling pathway, NK cell–mediated cytotoxicity, and T cell receptor signaling (Figure S3I), suggesting broad impairment of adaptive immune function.

### 3.4 Signatures of Interferon Response and Cytokines in Distinct Subtypes

**FIGURE 4:**
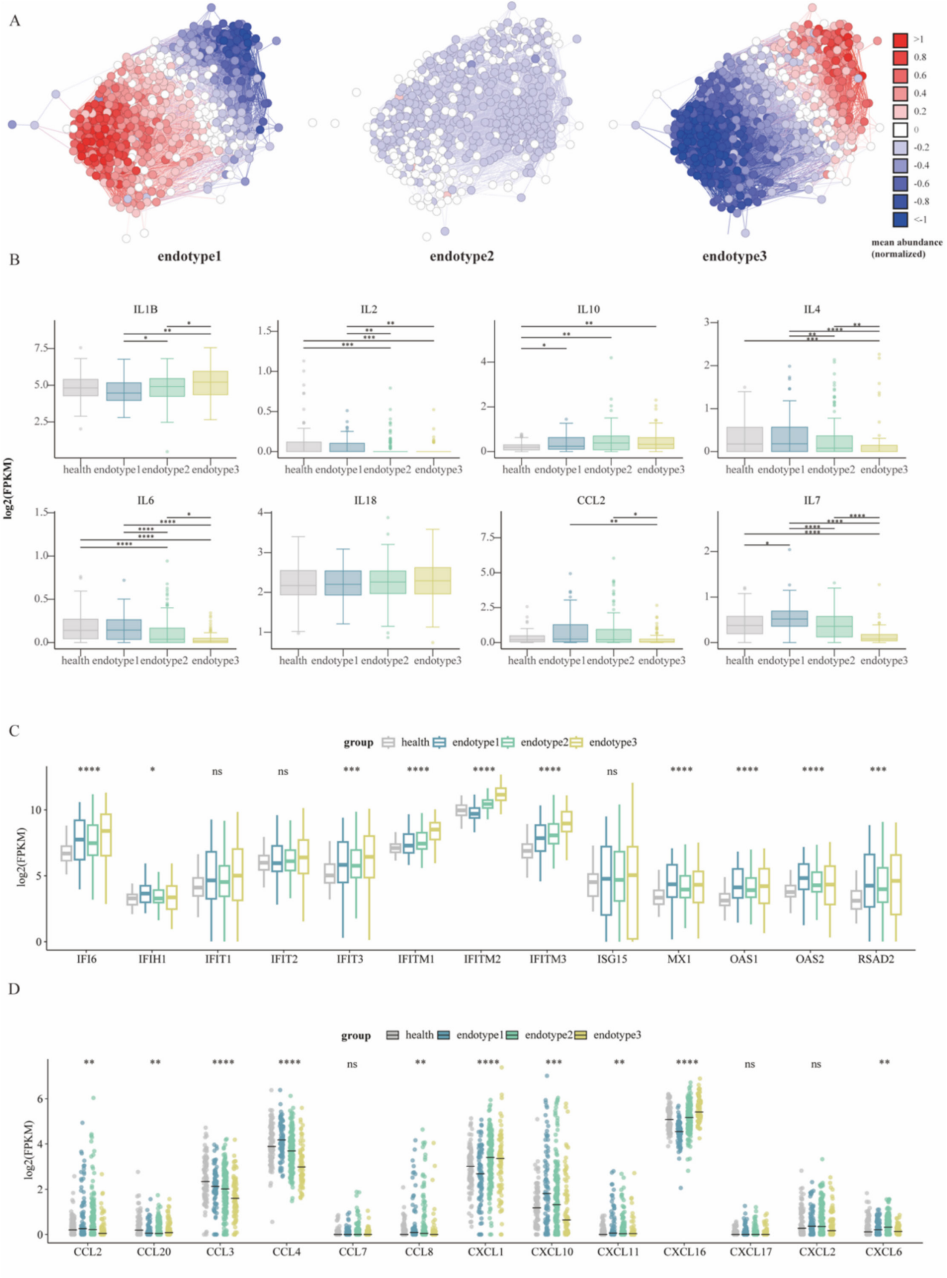
IFN and cytokine profiling in three subtypes. (A) IFN transcriptomics correlation network based on 590 ISGs. The average expression of genes in the correlation networks was presented. (B–D) Expression levels of cytokines (B), antiviral genes (C), and chemokines (D) across the three endotypes. Two‐sided *p* values were calculated using the Wilcoxon test for plot B and the Kruskal–Wallis test for plots C and D. *p* values from the Wilcoxon test were adjusted using the Benjamini–Hochberg procedure. The horizontal line within each box represents the median.

The IFN response plays a key role in SARS‐CoV‐2 infection and is associated with hyperinflammatory cytokine production, especially in severe cases. Biological function analysis revealed that the response to Type I interferon was upregulated across all endotypes (Figure 3C). This prompted us to further investigate the IFN features within different endotypes. ISG network analyses were conducted to explore endotype‐specific IFN features (Figure 4A). We found that genes were upregulated in endotype 3 but downregulated in endotype 1. Moreover, the expression of IFN‐associated cytokines showed that IL‐1B levels were markedly elevated in endotype 3, whereas the levels of IL‐2, IL‐4, CCL2, IL‐7, and IL‐6 were decreased. The levels of IL‐10 and IL‐18 remained similar across the three groups (Figure 4B). ISGs related to antiviral activity, including IFIT, OAS, and IFITM gene families, as well as other ISGs such as ISG15, RSAD2, and MX1, were analyzed (Figure 4C). Among them, the IFITM family genes showed increased expression across all three endotypes compared with healthy individuals, with the highest levels observed in endotype 3. In addition to the elevated production of pro‐inflammatory cytokines, severe COVID‐19 is characterized by increased expression of specific chemokines. For instance, elevated levels of the soluble CXCL16 (sCXCL16) chemokine have been reported in deceased COVID‐19 patients [33]. We also observed that CXCL16 is markedly upregulated in endotype 3, suggesting a role for CXCL16 in the pathogenesis of severe COVID‐19 (Figure 4D). Additionally, CCL2, CCL3, CCL4, and CXCL10 were higher in endotype 1.

### 3.5 Validation of the Predictive Model for Stratifying COVID‐19 Patients

**FIGURE 5:**
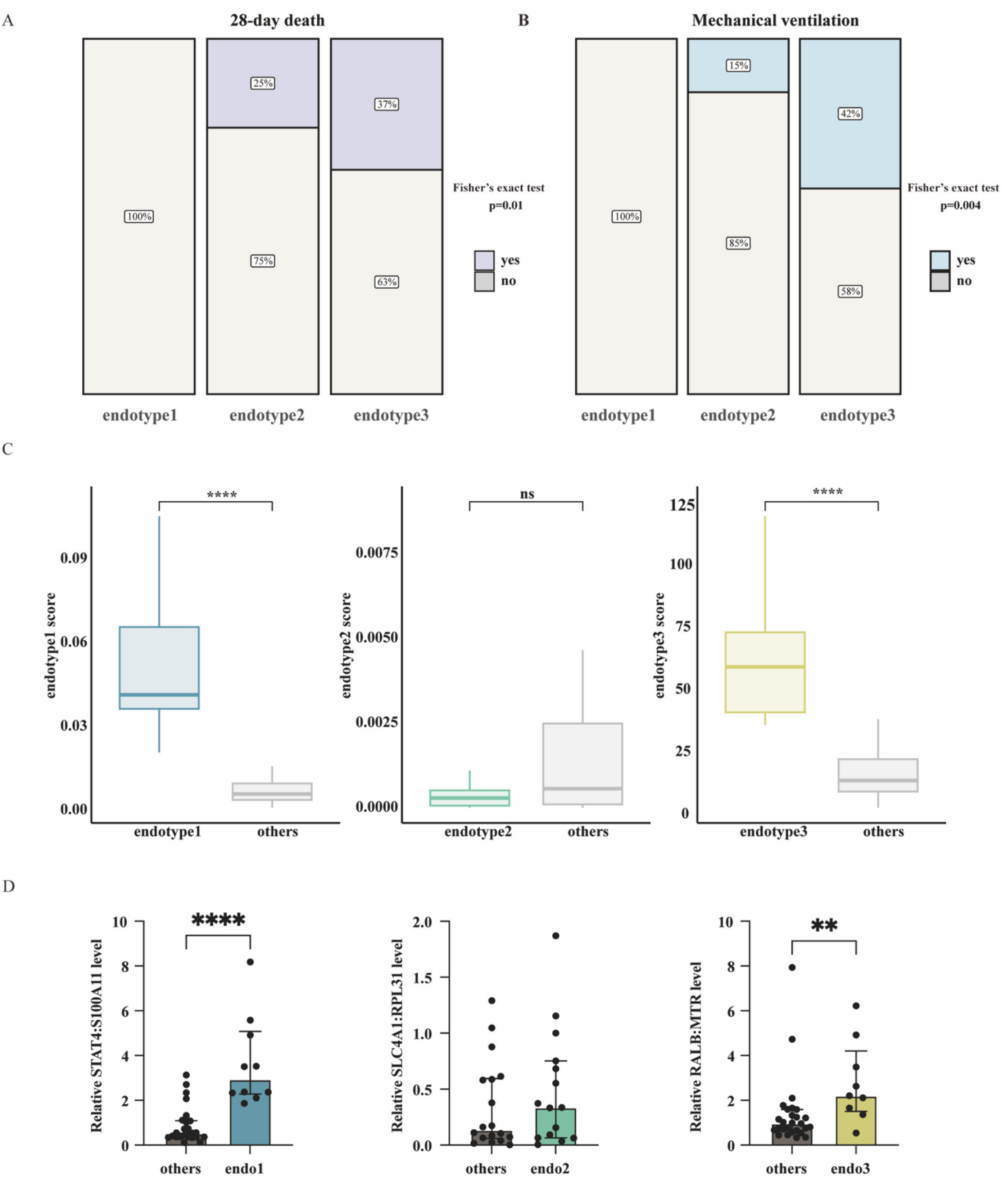
Assessment of COVID‐19 subtypes in the validation cohort. (A) 28‐day mortality proportions among different endotype groups. (B) Proportions of mechanical ventilation across endotype groups. (C) Expression levels of candidate endotype biomarkers (gene expression ratios). Two‐sided *p* values were obtained using the Wilcoxon test. (D) Relative mRNA expression levels of endotype biomarkers measured by qPCR. Data are presented as median ± interquartile range (IQR). Statistical significance was assessed using the two‐sided Wilcoxon test.

We next examined whether the predictive model reliably stratified patients in the two validation cohorts [34]. The random forest classifier classified patients into their endotype groups. Similar to the discovery cohort, patients in the endotype 3 group had the worst outcomes. At Day 28, 7 out of 19 patients (37%) with endotype 3 had died, compared to 5 out of 20 patients (25%) with endotype 2 (Figure 5A). The proportion of patients requiring mechanical ventilation was also highest in endotype 3 (42%) and lowest in endotype 1 (Fisher's exact test, *p* = 0.004) (Figure 5B). In the additional validation cohort GSE217948, mortality remained highest in endotype 3 (Fisher's exact test, *p* = 0.01), whereas ICU admission rates were similar across groups (*p* = 0.1, Figure S4A). The candidate biomarker models for endotypes accurately classified patients in the endotype 1 and endotype 3 groups but not in the endotype 2 group (Figures 5C and S4B). One possible reason for the reduced classification performance is that endotype 2 represents an intermediate or transitional state, leading to greater instability in its molecular signature. Moreover, the candidate biomarker was validated at the mRNA level using qPCR, supporting its potential clinical utility (Figure 5D). Collectively, these findings support the robustness of our predictive model and suggest that the identified candidate biomarkers have the potential to be broadly applied for the stratification of COVID‐19 patients. Next, we employed CIBERSORT to estimate the proportions of various immune cell types (Figure S4C). Patients in the endotype 3 group had higher fractions of monocytes in all cohorts. In contrast, the proportions of NK cells were lower in endotype 3. To explore whether specific immune cell populations may contribute to the transcriptional signatures defining each endotype, we next examined the correlations between the top endotype‐specific genes and the inferred immune cell proportions. Notably, OLAH, which was markedly upregulated in endotype 3, exhibited a positive correlation with monocytes proportion in this endotype (Figure S4D).

Using the validation cohort, we found that the cytokines IL‐1B and IL‐7 exhibited similar trends as those observed in the discovery cohort. Notably, IL‐10 was upregulated in the endotype 3 group of the validation cohort (Figure S5A,B). CRP, D‐dimer, and procalcitonin (PCT) tended to be highest in endotype 3 and lowest in endotype 1, consistent with patterns observed in the discovery cohort (Figure S5C). Altogether, these laboratory findings further indicate that the endotype 1 subtype of COVID‐19 was associated with the best outcomes, while the endotype 3 subtype had the worst outcomes.

## 4 Discussion

We identified three endotypes of COVID‐19 patients based on blood RNA expression profiles, each characterized by distinct clinical outcomes and biological features. These endotypes were significantly associated with clinical features: Patients classified as endotype 1 had better outcomes, whereas those in the endotype 3 group experienced the poorest prognosis. Abnormal laboratory measurements, such as elevated CRP, D‐dimer, and PCT, biomarkers linked to COVID‐19 severity, were highest in endotype 3, suggesting a dysregulated immune response in this group. From a biological perspective, the endotype 1 group exhibited an attenuated response to IL‐1 signaling, along with upregulated expression of IL‐7. The endotype 2 group showed enrichment in response to decreased oxygen levels and the angiotensin‐activated signaling pathway. The endotype 3 group was characterized by upregulated TLR4 signaling and increased IL‐1β expression, accompanied by suppressed NK cell–mediated cytotoxicity. Finally, to guide clinical practice, we developed predictive biomarker models for each endotype: STAT4:S100A11 for endotype 1, SLC4A1:RPL31 for endotype 2, and RALB:MTR for endotype 3. These predictive biomarker models were further validated in independent cohorts, demonstrating favorable performance.

Although antiviral therapy and immunomodulators have been approved for treating COVID‐19, the disease remains a significant public health concern. A major challenge in COVID‐19 clinical management is the lack of biomarkers to guide precision therapies. Previous studies have established classification models [7, 19, 35], such as CTP1, which is characterized by an IFN‐driven response and has been associated with worse outcomes compared to CTP2 [18]. However, these models require refinement for COVID‐19 due to the following reasons: (1) They fail to fully capture disease heterogeneity, (2) small sample sizes limit model accuracy, and (3) their clinical applicability remains challenging. To address these limitations, we leveraged publicly available transcriptional data from 351 patients to propose novel COVID‐19 subtypes and identify six genes as candidate biomarkers for their classification.

In addition to identifying transcriptomic differences among endotypes, our findings align with several previously reported immune signatures in severe COVID‐19. Endotype 3, which consistently showed the highest mortality across validation cohorts, was characterized by markedly increased expression of CD177, a neutrophil‐associated activation marker. Elevated CD177 expression has been repeatedly linked to severe COVID‐19 pneumonia and proposed as a potential prognostic indicator in prior studies [4, 7, 36]. Similarly, patients in endotype 3 exhibited reduced proportions of resting NK cells, a pattern consistent with the previously reported Im‐C1 immune subtype associated with unfavorable outcomes [19]. Together, these concordant observations support the notion that molecular endotype can delineate distinct immune response programs underlying the heterogeneity of COVID‐19.

Among these findings, OLAH was most prominently upregulated in endotype 3, consistent with previous reports linking OLAH to severe viral respiratory infection [37]. OLAH expression was predominantly detected in CD14^+^ monocytes [37] and showed a strong positive correlation with monocyte proportions within endotype 3. These results suggest that monocytes may be key contributors to the transcriptional signature characterizing endotype 3. Additionally, the elevated monocyte proportions observed in this endotype raise the possibility that peripheral monocyte levels may reflect the underlying inflammatory state and could potentially serve as an accessible blood‐based indicator of disease severity in COVID‐19, although further prospective studies are warranted.

Using consensus clustering, we identified clinically meaningful stratification of COVID‐19 patients based on immune transcriptomic profiles, similar to other diseases [21, 38, 39, 40]. This classification provides novel insights into the immune dysregulation underlying severe SARS‐CoV‐2 infection. Patients in the endotype 3 group, who had the worst clinical outcomes, exhibited elevated IL‐1β expression. Conversely, IL‐7 expression was downregulated in endotype 3. IL‐7 is a growth and antiapoptotic cytokine with potent survival and proliferative activity during both B and T lymphopoiesis [41, 42]. Lymphopenia is frequently observed in patients with severe COVID‐19 and has been associated with increased mortality [43, 44]. These cytokine expression patterns highlight distinct immune dysregulation across molecular endotypes.

This study has several limitations. The current conclusions are primarily derived from publicly available datasets and two relatively small validation cohorts. Therefore, further validation in larger, independent prospective cohorts is essential to confirm our findings and assess their generalizability. Moreover, the reduced classification performance observed for endotype 2 may be explained by its biological nature, as this group appears to represent an intermediate or transitional transcriptional state, leading to greater heterogeneity and lower classification stability across datasets. In addition, the datasets incorporated in this study differ in sample types (PBMCs, whole blood, and leukocytes) and clinical severity. Such heterogeneity may introduce potential confounding effects, particularly those arising from differences in underlying cell‐type composition. Finally, the proposed cutoff values and gene‐ratio thresholds should be interpreted as hypothesis‐generating and will require further optimization and rigorous validation prior to clinical use. Future work should validate our biomarker models in large, independent, multicenter prospective cohorts to confirm performance, refine threshold calibration, and assess generalizability across clinical settings. Importantly, these prospective studies should also evaluate clinical utility to clarify a feasible path toward implementation.

The molecular classification was provided for patients with COVID‐19. Our integrated transcriptional data enhance the understanding of heterogeneity and underlying pathogenetic mechanisms in COVID‐19 by defining patient subgroups. Furthermore, we propose biomarker models to facilitate patient stratification and personalize therapies.

## Author Contributions

**Hongyu Liu:** conceptualization, methodology, visualization, validation, formal analysis, datacuration, writing – original draft, writing – review and editing. **Ying Zheng:** investigation, writing – review and editing, methodology. **Xiaoyan Deng:** conceptualization, formal analysis, writing – review and editing. **Mengxue Li:** formal analysis. **Di He:** visualization. **Wenting Zuo:** visualization. **Yitian Xu:** writing – review and editing. **Xuhui Shen:** writing – review and editing. **Haibo Li:** conceptualization, supervision, writing – review and editing. **Bin Cao:** conceptualization, funding acquisition, project administration, supervision. All authors reviewed the manuscript, agreed to its publication, and had full access to all study data. They also approved the final version of the manuscript.

## Funding

This work is supported by the National High Level Hospital Clinical Research Funding (2025NHLHCRF‐JBGS‐B‐WZ‐05), National Natural Science Foundation of China (82470007, 82530002), Beijing Research Ward Excellence Program (BRWEP2024W114060103), Chinese Academy of Medical Sciences Innovation Fund for Medical Sciences (2021‐I2M‐1‐048), New Cornerstone Science Foundation, Chinese Academy of Medical Sciences Innovation Fund for Medical Sciences (2023‐I2M‐2‐001), State Key Laboratory Special Fund (2060204), National Key Research and Development Program of China (2021YFC2300501), and Noncommunicable Chronic Diseases‐National Science and Technology Major Project (2023ZD0506200, 2023ZD0506203).

## Conflicts of Interest

The authors declare no conflicts of interest.

## Supporting information


**Figure S1:** Consensus clustering.
**Figure S2:** DEGs and performance of machine learning methods.
**Figure S3:** Construction of the WGCNA co‐expression network and functional enrichment analysis.
**Figure S4:** COVID‐19 subtypes and immune cell proportions in the validation cohort.
**Figure S5:** Cytokine expression across endotypes in the validation cohort.
**Table S1:** Information of seven GEO datasets.
**Table S3:** Demographic information of the validation cohort.
**Table S4:** Primer sequences used for quantitative real‐time PCR.

**Table S2:** PC1_Loadings_and_Enrichment.

## Data Availability

The data that support the findings of this study are available from the corresponding author upon reasonable request.
