# Supplementary material for: Molecular Classification of Patients With COVID‐19 Based on Transcriptional Profiling
Source: Influenza Other Respir Viruses. 2026 Feb 10;20(2):e70227. doi: 10.1111/irv.70227 (PMC12887440; doi:10.1111/irv.70227)
Supplement: Supplementary file 1 — Figure S1: Consensus clustering. Figure S2: DEGs and performance of machine learning methods. Figure S3: Construction of the WGCNA co‐expression network and functional enrichment analysis. Figure S4: COVID‐19 subtypes and immune cell proportions in the validation cohort. Figure S5: Cytokine expression across endotypes in the validation cohort. Table S1: Information of seven GEO datasets. Table S3: Demographic information of the validation cohort. Table S4: Primer sequences used for quantitative real‐time PCR. [file IRV-20-e70227-s002.docx]

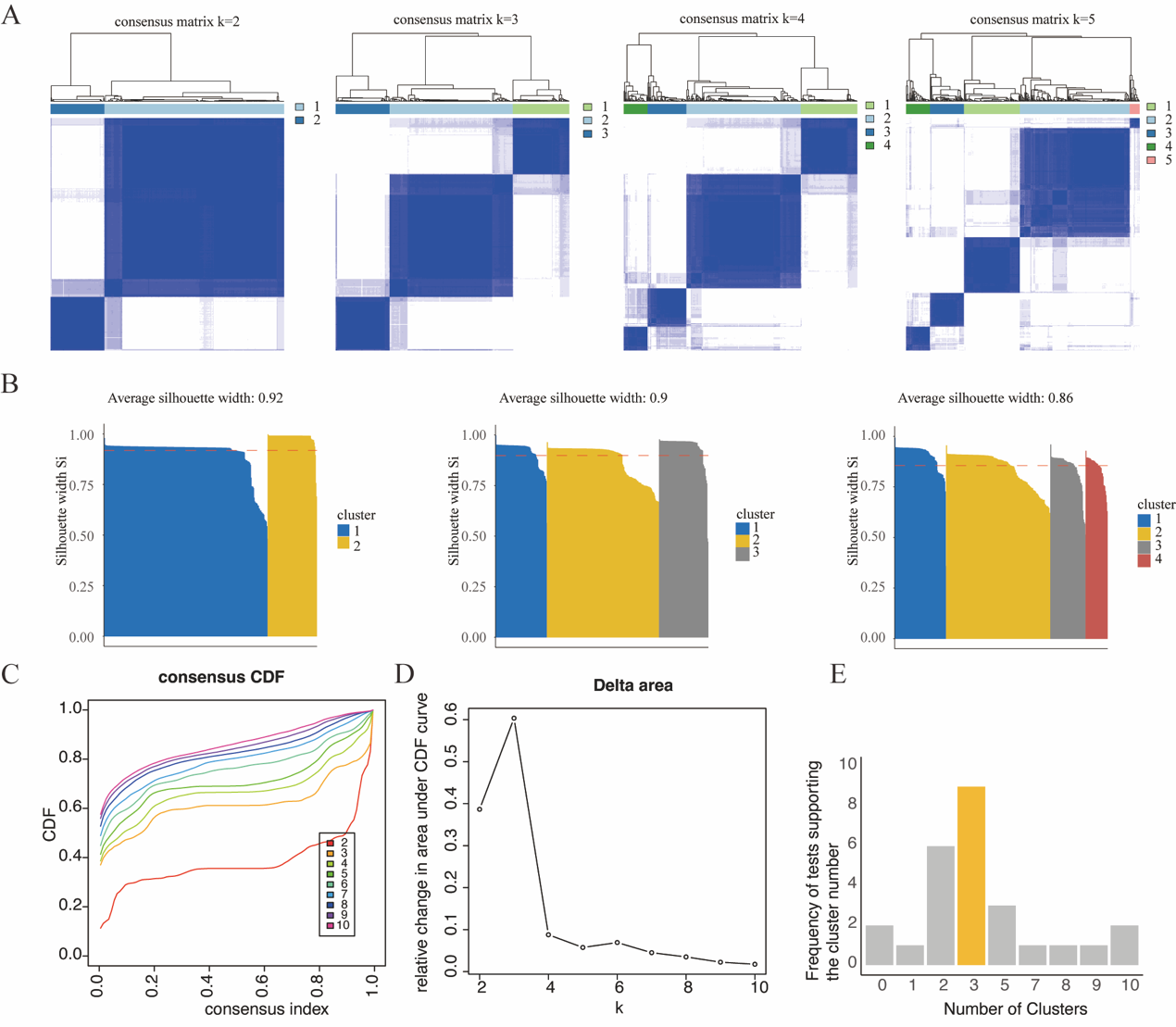


**Fig S1** **Consensus clustering**

(A) Unsupervised consensus clustering of identified subtypes (k =2 to 5).

(B) Silhouette width analysis indicating stable partitioning into three subtypes.
(C) The cumulative distribution function (CDF) curves

(D) The relative change in the area under the CDF curve (Delta area)

(E) Bar plot showing the optimal number of clusters determined using the NbClust R package.


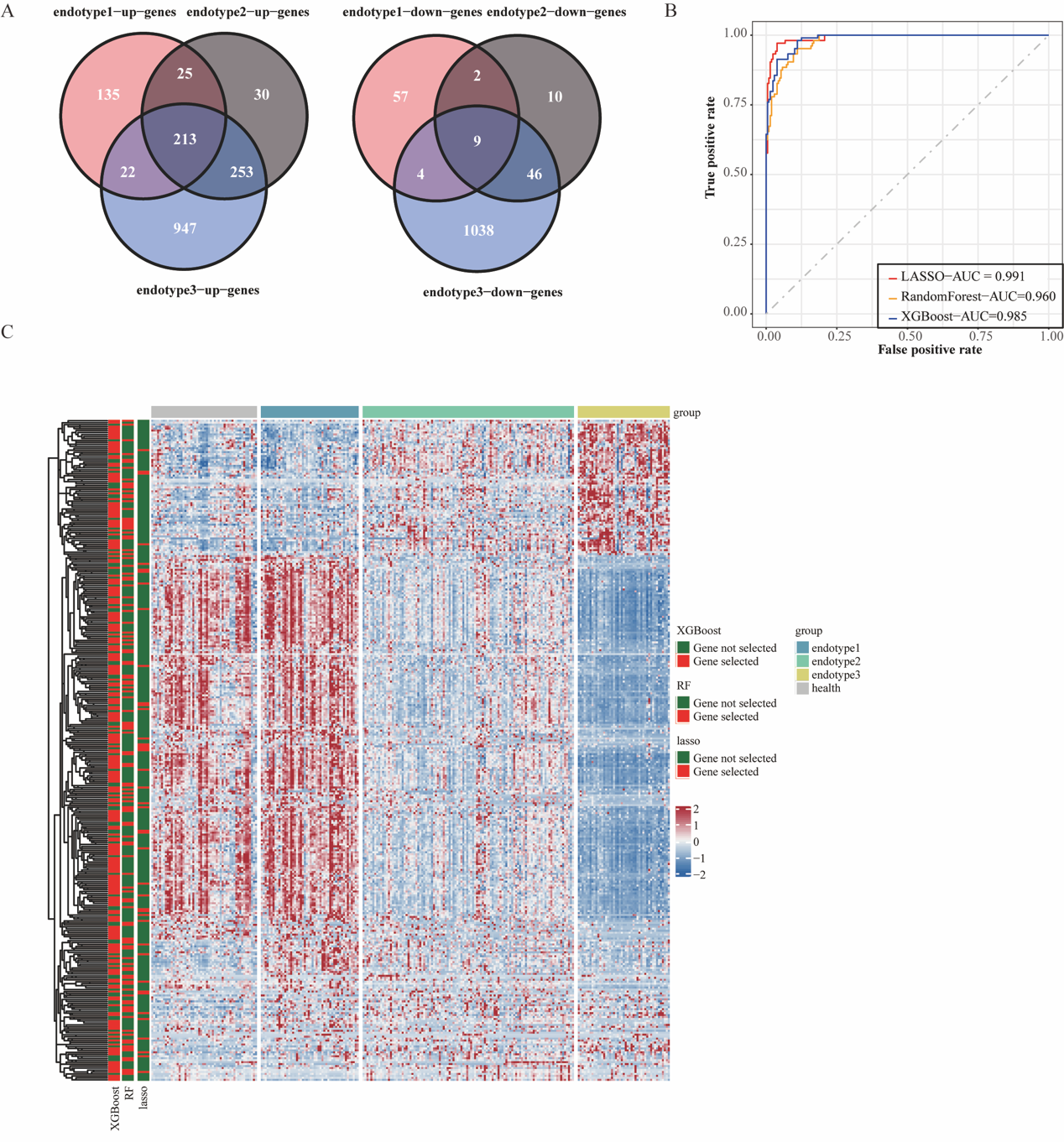


**Fig S2 DEGs and performance of machine learning methods**

(A) The Venn diagram shows the intersection of the differentially expressed genes (DEGs) in the endotype 1–3 groups.

(B) The performance of three machine learning methods.

(C) All DEGs selected by three machine learning algorithms.


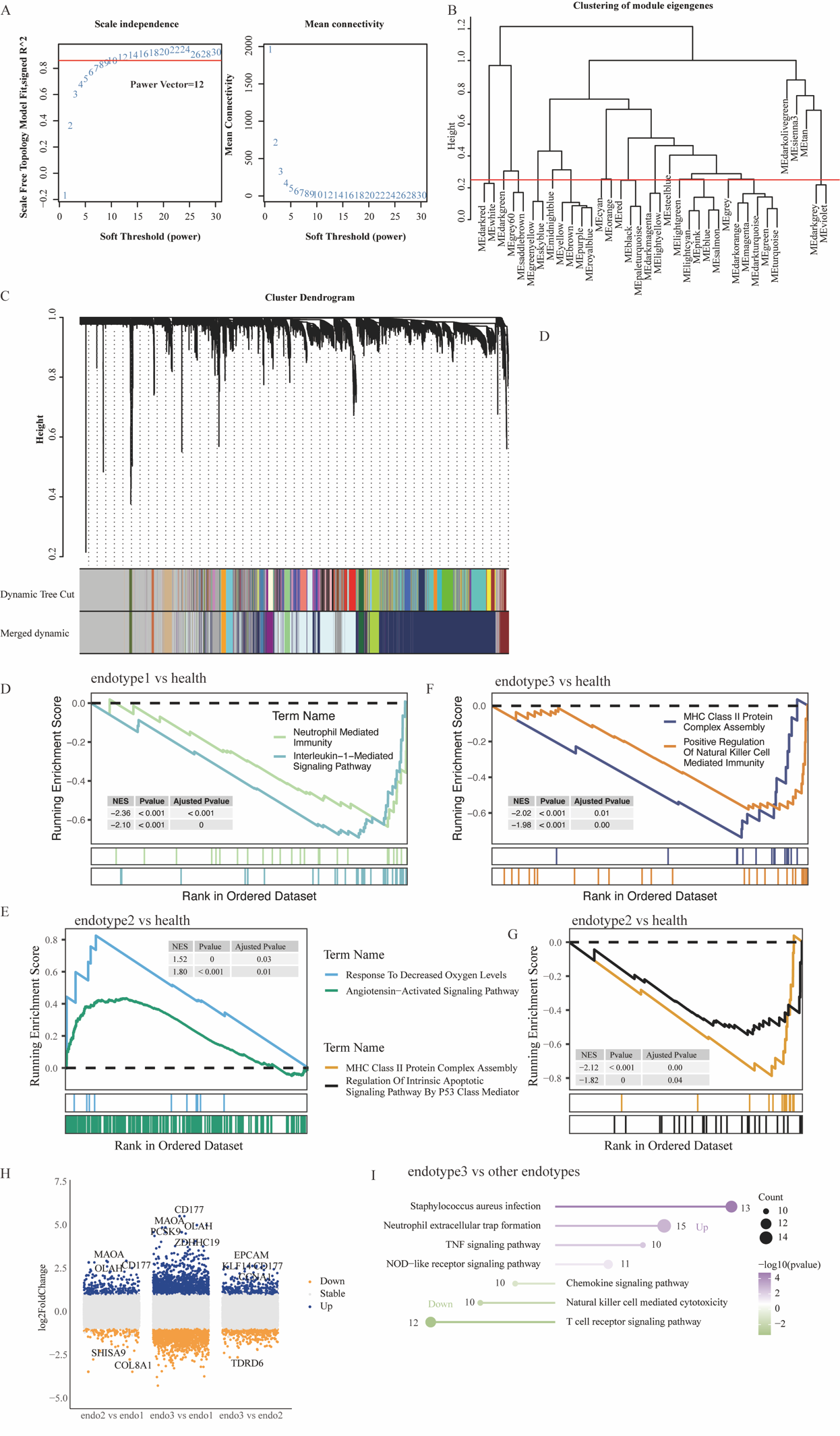


**Fig S3 Construction of the WGCNA co-expression network and functional enrichment analysis**

(A) Determination of the optimal soft-thresholding power for network construction. A soft threshold of β = 12 was selected.

(B) Module dendrogram showing merging of highly similar modules after cutting the tree at a height of 0.25.

(C) Gene clustering dendrogram of the top 50% (8,471) most variable genes, based on dissimilarity calculated from 1 − TOM.

(D-G) Representative GSEA enrichment plots (GO gene sets) illustrating differential immune responses in each endotype compared with healthy controls. Panels show enrichments for endotype 1 (D) and endotype 2 (E, G), as well as endotype 3 (F).

(H) Volcano plot showing differentially expressed genes across the three endotypes.

(I) KEGG pathway enrichment analysis of endotype 3 compared with the other endotypes. The line represents the -log10(P value), and the dots indicate the number of genes enriched in each pathway. A P value < 0.05 was considered statistically significant.

**
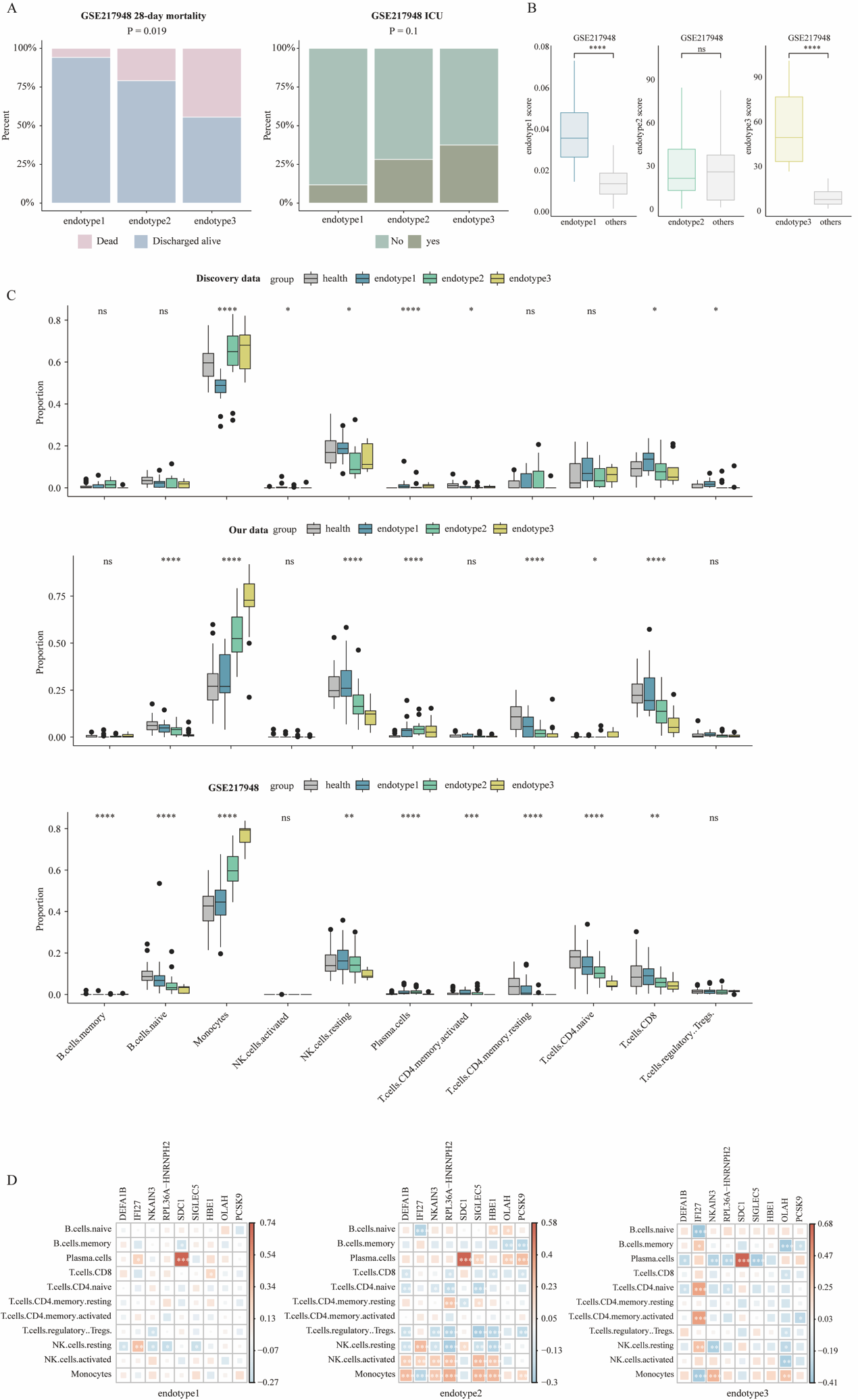


Fig S4. COVID-19 subtypes and immune cell proportions in the validation cohort.**

(A) Proportions of 28-day mortality and ICU admission among different endotype groups in GSE217948. P-value were obtained using the Fisher’s exact test.

(B) Expression levels of candidate endotype biomarkers (gene expression ratios) in GSE217948. Two-sided p-value were obtained using the Wilcoxon test.

(C) Proportions of various immune cell types in the discovery cohort (PBMC samples from GSE152418 and GSE179627), in our cohort, and in GSE217948. Two-sided p-values were calculated using the Kruskal–Wallis test.

(D) Correlation heatmap showing the associations between the top endotype-specific genes and the inferred immune-cell proportions.


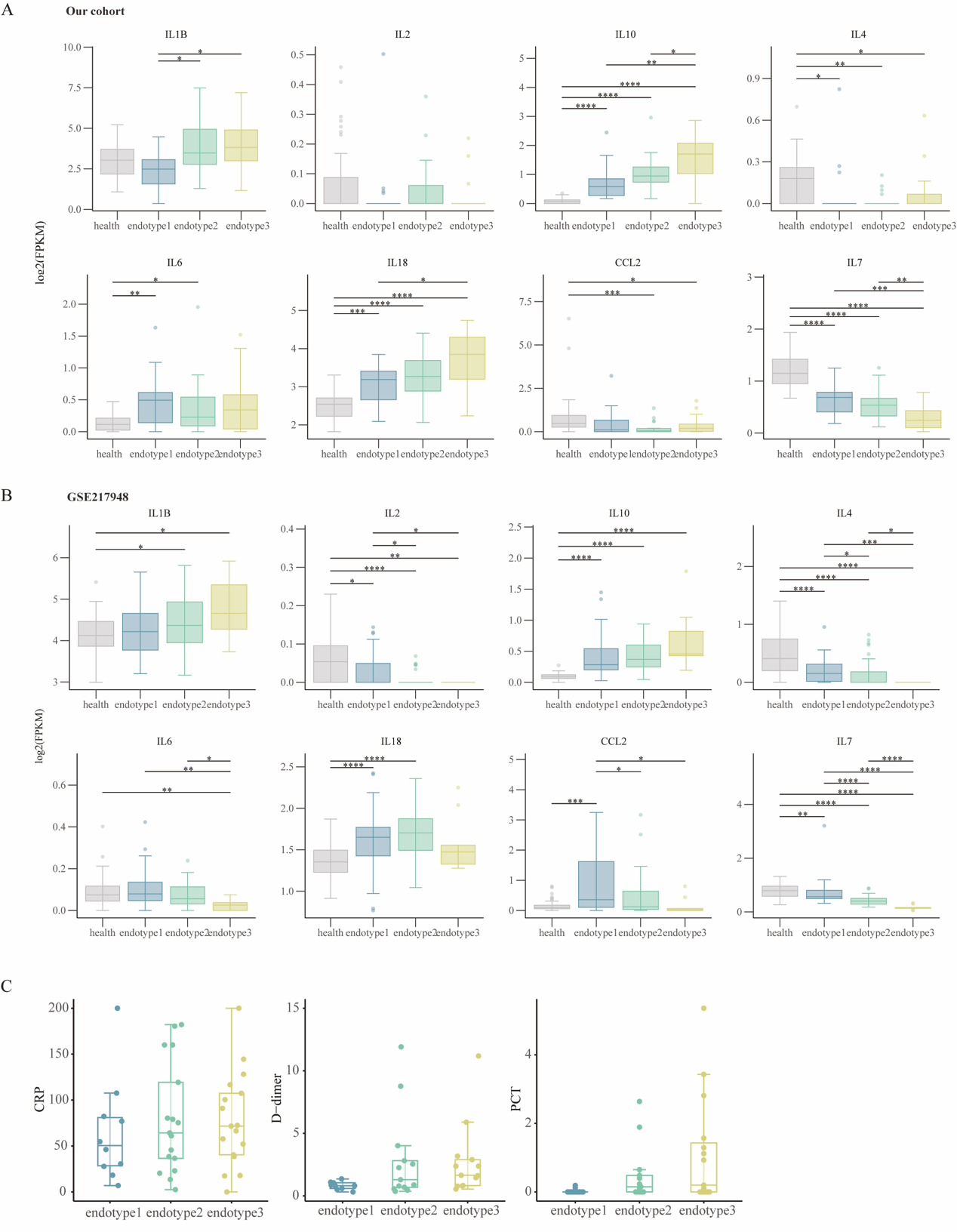


**Fig. S5. Cytokine expression across endotypes in the validation cohort.**

(A-B) Expression levels of cytokines in the two validation cohorts: our cohort (A) and GSE217948 (B). Two-sided p-values were calculated using the Wilcoxon test and adjusted using the Benjamini–Hochberg procedure.

(C) Comparisons of laboratory measurements (CRP, D-dimer, and PCT) in the validation cohort. Two-sided p-values were calculated using the Wilcoxon test and adjusted using the Bonferroni correction.

**Table S1**

Information of seven GEO datasets

| Dataset | Platform | COVID-19/Health | Sample | Disease severity | Endotype |
| --- | --- | --- | --- | --- | --- |
| GSE152418 | GPL24676 | 16/17 | PBMC | Moderate: 4 (25%) Severe (no ICU): 8 (50%) ICU: 4 (25%) | endotype1: 6 (38%)  endotype2: 6 (38%)  endotype3: 4 (25%) |
| GSE152641 | GPL24676 | 62/24 | Whole blood | Intubation: 23 (37%) | endotype1: 17 (27%)  endotype2: 32 (52%)  endotype3: 13 (21%) |
| GSE157103 | GPL24676 | 100/0 | Leukocyte | ICU: 50 (50%)  Non-ICU: 50 (50%) | endotype1: 25 (25%)  endotype2: 44 (44%)  endotype3: 31 (31%) |
| GSE161731 | GPL24676 | 77/19 | Whole blood | Hospitalized: 12 (16%)  Non-Hospitalized: 65 (84%) | endotype1: 8 (10%)  endotype2: 59 (77%)  endotype3: 10 (13%) |
| GSE171110 | GPL16791 | 44/10 | Whole blood | ICU or transfer to ICU or death: 36 (82%) | endotype1: 13 (30%)  endotype2: 18 (41%)  endotype3: 13 (30%) |
| GSE179627 | GPL24676 | 21/22 | PBMC | Asymptomatic: 8 (38%) Symptomatic: 13 (62%) | endotype1: 9 (43%)  endotype2: 8 (38%)  endotype3: 4 (19%) |
| GSE222393 | GPL16791 | 31/0 | Whole blood | ICU: 21 (100%) | endotype1: 7 (23%)  endotype2: 18 (58%)  endotype3: 6 (19%) |

|  | **Total N=56** | **endotype1 N=17** | **endotype2 N=20** | **endotype3 N=19** | **P** |
| --- | --- | --- | --- | --- | --- |
| **Demographics** |  |  |  |  |  |
| Sex, male, % | 41 (73) | 11 (65) | 15 (75) | 15 (79) | ns |
| Age,Median(IQR),yrs | 73 (68-82) | 73 (70-80) | 74 (63-82) | 73 (67-86) | ns |
| **Comorbidity (%)** |  |  |  |  |  |
| Hypertension | 30 (54) | 11 (65) | 10 (50) | 9 (47) | ns |
| Diabetes | 19 (34) | 3 (18) | 8 (40) | 8 (42) | ns |
| Malignancy | 5 (9) | 1 (6) | 1 (5) | 3 (16) | ns |
| Cerebrovascular disease | 4 (7) | 0 (0) | 1 (5) | 3 (16) | ns |
| COPD | 2 (4) | 0 (0) | 1 (5) | 1 (5) | ns |
| Renal insufficiency | 5 (9) | 1 (6) | 2 (10) | 2 (11) | ns |
| Chronic liver disease | 1 (2) | 1 (6) | 0 (0) | 0 (0) | ns |
| Cardiovascular compromise | 12 (21) | 6 (35) | 2 (10) | 4 (21) | ns |
| **Outcome** |  |  |  |  |  |
| Mechanical ventilation, % | 11 (20) | 0 (0) | 3 (15) | 8 (42) | 0.004 |
| ICU admission, % | 11 (20) | 1 (6) | 4 (20) | 6 (32) | ns |

**Table S3**Demographic Information of the Validation Cohort

The Kruskal-Wallis test was used for continuous data, while the Chi-square test or Fisher's exact test was applied for categorical data.

**Table S4**Primer sequences used for quantitative real-time PCR

| **Gene symbol** | **Forward primer (5'–3')** | **Reverse primer (5'–3')** |
| --- | --- | --- |
| STAT4 | TAAAGGCCGGTTGTCTGCTC | GCAAGGCTGAGAGCTGTAGT |
| MTR | AGCGGGAGAAGCTAAACGAAG | CGGTAGGCCAAGTGTTCAAGG |
| RPL31 | CTCGGGCACTCAAAGAGATTC | CGGATTCGGTATGGCACATTC |
| S100A11 | CTGAGCGGTGCATCGAGTC | TGTGAAGGCAGCTAGTTCTGTA |
| SLC4A1 | GGTGATGGACGAAAAGAACCA | AAGACTCTACGCAGCTCTAGG |
| RALB | AGCCCTGACGCTTCAGTTC | AGCGGTGTCCAGAATATCTATCT |
| ACTB | CATGTACGTTGCTATCCAGGC | CTCCTTAATGTCACGCACGAT |
